# The temporal dynamics of perceived stress and depression in college students: An ecological momentary assessment

**DOI:** 10.1002/pchj.803

**Published:** 2024-10-04

**Authors:** Guo Feng, Xiaxia Xu, Jie Wu, Jiawei Lei

**Affiliations:** ^1^ Psychological Research and Counseling Center Southwest Jiaotong University Chengdu Sichuan China; ^2^ Institute of Applied Psychology Southwest Jiaotong University Chengdu Sichuan China

**Keywords:** depression, ecological momentary assessment, perceived stress, state rumination, trait rumination

## Abstract

Previous studies have implied that stress is a risk factor for depression, but relatively little is known about how healthy individuals' stress dynamically affects depression as a mood in daily life. Therefore, an ecological momentary assessment study was conducted among 141 college students to test the temporal dynamic effect of daily perceived stress on depression and the underlying mediating and moderating role of rumination. Perceived stress, state rumination, and depression were measured using self‐compiled questionnaire three times a day over 12 days. Trait rumination was measured with the Nolen‐Hoeksema Ruminative Response Scale. Hierarchical linear models with HLM 7.0 were adopted to examine the mediation and moderation effects. In the mediation model, the greater the daily perceived stress at time *t*, the higher the state rumination at time *t* + 1, and state rumination at *t* + 1 positively predicted college students' depression at *t* + 2. In the moderation model, trait rumination significantly facilitated perceived stress‐induced depression. These results verified that daily perceived stress could affect college students' depression directly or indirectly through the critical mediating mechanism of state rumination, and this effect would be exacerbated with a higher level of trait rumination.

## INTRODUCTION

College students face many changes in the transition from late adolescence to emerging adulthood that lead to various daily pressures (Reyes‐Rodríguez et al., 2013) and an exceptionally high prevalence of depression (Fawzy & Hamed, 2017). Numerous studies have inquired into the impact of stress on depression (LeMoult et al., 2020). However, these studies have mostly focused on the relationship between accumulated pressure and depression, and relatively few have explored the dynamic effects of subtle stress perception and depression as a mood in daily life. Hence, both longitudinal and cross‐sectional studies have generally regarded stress and depression as relatively “stable” states and used retrospective questionnaires to measure them, which may not accurately reflect the real situation of individuals due to retrospective bias (Van Den Brink et al., 2001). However, individuals' stress and depression are closely related to their life situation at that time. For each individual, how does stress dynamically affect their depressive mood in daily life? This is a question that has not been fully explored. To investigate this problem, the present study aimed to adopt the dynamic assessment method (Trull & Ebner‐Priemer, 2014) to track and measure stress and depression in daily life continuously.

### Daily perceived stress and depression

Perceiving excessive stress may lead to severe physical and psychological problems, including depression—a significant period of low mood or anhedonia that interferes with daily functioning (Piccolo & Noble, 2018). The diathesis‐stress model (Monroe & Simons, 1991) and empirical studies have posited and demonstrated that perceived stress is a significant predictor of depression (Gavurova et al., 2020).

However, prior studies have mostly considered the causes of depression from the perspective of stressors (Kira et al., 2021) or stress coping styles (Sempértegui et al., 2017), while few have studied the formation mechanism of depression from the perspective of stress perception. Every individual will encounter some form of stress, but their “internal cognition” of such an “external” event varies (Kemeny, 2003). The cognition of stress is different, as is the impact of stress on individuals. College students who perceive more stress are more likely to experience negative emotions, including depression, in response to stressful events. Furthermore, despite empirical studies that have shown that stress perception has a positive predictive effect on college students' depression (Gavurova et al., 2020), scarce information exists about how perceived stress in daily life can subtly lead to depressive symptoms, especially for nondepressed healthy college students. During the college phase, young adults socialize outside of the family and school, engaging with society where they encounter numerous acute and chronic stressors, which have been proven to be a vital factor for college students to suffer from depression (Wang et al., 2024). A mental health survey in China revealed that the highest detection rate of depression was 24.1% in the 18‐ to 24‐year‐old age group, primarily represented by college students (Fu et al., 2022). Therefore, depression prevention and intervention studies among college students are still urgent. The current study focuses on the nondepressed college student population because nondepressed college students constitute a broader population, which means that a large group of people will benefit from the present study. Furthermore, early prevention strategies focused on individuals who are not clinically diagnosed with depression make it possible to reduce the incidence of depression and related problems, allowing mental health resources to be effectively allocated and utilized. Therefore, to better reduce and prevent depressive symptoms in healthy college students, it is necessary to investigate the association between daily perceived stress and depression from the perspective of short‐term dynamic variability.

### State rumination as a mediator

Although studies have shown that stress perception may impact individuals' depression, the mediating and moderating mechanism of the impact is unclear. Stress perception is the psychological response of individuals to threatening stimuli following cognitive evaluation (Godoy et al., 2018), which may closely relate to rumination if the cognitive evaluation is negative and occurs repeatedly. Rumination refers to a form of negative repetitious thoughts in which individuals concentrate on the reasons and repercussions of a distressing circumstance or occurrence (Grafton et al., 2016). Research studies have shown that the relationship between rumination and stress is complex and bidirectional. On the one hand, individuals who ruminate more are more likely to experience stress due to their inability to disengage from negative thoughts, which can intensify the perception of stressors (Michl et al., 2013). On the other hand, stress itself can trigger ruminative thoughts, creating a cycle that sustains and amplifies psychological distress (Michl et al., 2013; Watkins & Roberts, 2020). In the present study, we treated rumination as a variable subsequent to stress. Despite being mostly treated as a stable trait‐like construct, rumination in daily life is an ongoing dynamic process (Marchetti et al., 2018). Individuals with more perceived stress tend to engage in more state rumination. For instance, a study indicated that women with human immunodeficiency virus (HIV) who perceive daily stress report more rumination about their bodily states (Millon & Shors, 2021). Therefore, it is reasonable to presume that people with a higher level of stress perception tend to use more ruminative thinking.

A growing number of studies have revealed that rumination is a significant predictor of depression (Fang et al., 2019; Fowler et al., 2017). Stress‐reactive theory postulates that stress‐induced rumination manifests as repetitive thinking of inferences about stressful events, in combination with negative cognitive style, and can eventually lead to depression (Rood et al., 2010). In line with the theoretical framework, a study has proved that rumination about stressful events is linked to higher depressive symptoms in adolescents (Fowler et al., 2017). Additionally, in an ecological momentary assessment (EMA) study, rumination at time *t* is positively associated with depression scores at time *t* + 1 (Fang et al., 2019). Thus, the present study proposed state rumination as a potential mediator between daily perceived stress and depression.

### Trait rumination as a moderator

Although daily perceived stress is associated with depression, not all healthy people with daily perceived stress suffer from depression. The heterogeneity in results may stem from personality characteristics that moderate the effect of daily perceived stress on depression, such as the trait of rumination (Skitch & Abela, 2008). In contrast to state rumination, trait rumination is a stable variable, which is defined as the tendency to repeatedly engage in negative thoughts and has been shown to lead to serious maladaptation (Whitmer & Gotlib, 2013). A few studies have examined trait rumination as a moderator to explore the potential conditional effects between stressors and mental health outcomes. For example, Bucknell et al. (2022) found that trait rumination moderates the association between adaptive self‐reflection and insight in the population of Australian Protestant Ministry workers. Similarly, Mezo and Baker (2012) collected a cross‐sectional sample of college students and demonstrated that the strength of the relationship between stress and depressive symptoms is moderated by the level of trait rumination among women. Specifically, this relationship is intensified with higher levels of trait rumination.

Despite evidence primarily derived from non‐intensive longitudinal data sources (e.g., cross‐sectional, longitudinal panel data), it is preliminarily assumed that trait rumination could work as a moderator, strengthening the effect between risky triggers and mental health conditions. The resource allocation hypothesis may provide a suitable explanation for the potential moderating role of trait rumination, postulating that individuals with rumination traits are more frequently in a state of spontaneous ruminative thinking (Connolly et al., 2014), which leads to a more serious depletion of cognitive resources and impairs executive control function, further increasing their likelihood of falling into depression (Watkins, 2002). From this, we deduce that college students with high levels of trait rumination are prone to cognitive resource deficits and find it challenging to deal with daily perceived stress, executive function impairment, and the increased risk of depressive development.

Another theory also supports the moderating role of trait rumination. The attention scope model of rumination suggests that a tendency to ruminate is associated with difficulties in updating working memory (WM) and disengaging from and forgetting no‐longer‐relevant information (Whitmer & Gotlib, 2013). When college students with high levels of trait rumination face daily stress, they may become fixated on negative information, associated with reinforced feelings of helplessness, thus predisposing them to depression (Cooney et al., 2010; Mandel et al., 2024). Thus, we assume that trait rumination could moderate the dynamic direct effect of perceived daily stress on depression among college students.

### The current study

This study aimed to test the temporal dynamic impact of perceived daily stress on depression and the underlying mediating and moderating role of rumination. We adopted an EMA design for data collection and applied a multilevel linear model to analyze the dynamic effects between variables in a natural context. The EMA design is suitable for assessing variables that are vulnerable to time, including stress perception, depression, and state rumination fluctuation, as the momentary data are typically collected several times a day for several days; compared to traditional methods, it has lower recall bias and higher ecological validity (Trull & Ebner‐Priemer, 2014).

**FIGURE 1:**
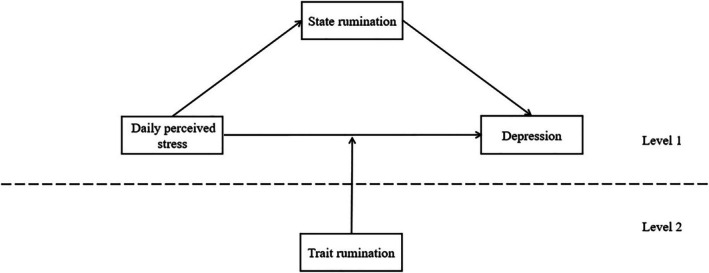
A cross‐level mediation moderation model of hypothesis.

Overall, we hypothesized that (1) daily perceived stress at time *t* would positively predict more depression at time *t* + 2, (2) state rumination at time *t* + 1 would mediate the dynamic effect of daily perceived stress at time *t* on depression at time *t* + 2, and (3) trait rumination would moderate the direct effect between daily perceived stress at time *t* and depression at time *t* + 2 (see Figure 1).

## METHODS

### Participants

All participants were randomly recruited through social media (WeChat and QQ) and posters at a Chinese university. The final sample comprised 141 undergraduate students (58% female) with a mean age of 20.10 years (*SD* = 1.63). All 141 participants achieved over 75% completion in the EMA. In the moderating effect analysis, 16 participants were excluded due to incomplete answers for the trait rumination scale, and the mean age of the remaining 125 participants (57% female) was 20.10 years (*SD* = 1.62). The Ethics Committee of Southwest Jiaotong University approved this study. All participants declared no history of mental disorders and provided written informed consent.

Participants first completed the Nolen‐Hoeksema Ruminative Response Scale (RRS; Nolen‐Hoeksema, 1991). Over the next 12 days, they were asked to complete a brief questionnaire accessed via a WeChat link (a Chinese messaging platform) on participants' mobile phones three times a day (at 9:30, 15:30, and 22:30). All participants were asked to complete the questionnaire within 1 h of receiving the link. The questionnaire contained measures of current perceived stress, depression, and rumination and could be completed within approximately 1 min. Participants had the opportunity to acquire 1–2 yuan after each submission.

### Measures

#### Interindividual variable

The interindividual variable was trait rumination assessed by the Nolen‐Hoeksema RRS. The 22‐item scale has been proven reliable and valid among Chinese samples (Han & Yang, 2009). Items were scored on a Likert‐type scale from 1 (*never*) to 4 (*always*), with higher scores indicating a higher degree of rumination. In this study, the reliability (Cronbach's *α*) coefficient was .94.

#### Intraindividual variables

In the current study, daily perceived stress, depression, and state rumination were intraindividual variables that needed to be measured multiple times.

Following Xu et al.'s (2017) research, daily perceived stress was measured using three self‐compiled items: (1) feeling unable to control important things in one's own life, (2) feeling nervous and stressed, and (3) constantly thinking things must be done by oneself. Items were rated on a Likert‐type scale from 0 (*not at all*) to 100 (*extremely*). The Cronbach's *α* of the questionnaire in this study was .83.

Depression was measured using one item: “Are you depressed at this moment?” This item was specifically compiled for the EMA in this study and was rated on a Likert‐type scale from 0 (*not at all*) to 100 (*extremely*), with higher scores manifesting more severe depression.

State rumination was also measured by a specifically developed item: “At this moment, I am thinking about my shortcomings, failures and mistakes in daily life.” It captured rumination thinking in one's momentary feelings. This item was also rated from 0 (*not at all*) to 6 (*extremely*), with higher scores indicating greater rumination.

### Data analysis

Hierarchical linear models have been increasingly used in EMA studies to explore the mediating effect of state variables and the moderating effect of trait variables (Du et al., 2019; Xu et al., 2017). Hence, we initially constructed a 1‐1‐1 multilevel mediation model to test our mediation hypotheses. At Level 1 of this model, the independent, mediating, and dependent variables were daily perceived stress_*t*_, state rumination_*t*+1_, and daily depression_*t*+2_, respectively. Following the multilevel mediation approach of “centered within the context with the reintroduction of the subtracted means,” we added the means of state variables for each participant (overall perceived stress, overall rumination) as independent and mediating variables to Level 2 (interindividual). The causal steps approach (Baron & Kenny, 1986) was used to test the mediation process between daily perceived stress_*t*_ and depression_*t*+2_. Subsequently, we examined the moderating effect of trait rumination on the relationship between daily perceived stress and depression. In this analysis, trait rumination was added to Level 2 of the model as a predictive variable for the regression coefficient of daily perceived stress_*t*_ on depression_*t*+2_ at Level 1. All data analyses were performed in HLM 7.0. The detailed formulae are presented in the Supporting Information.

## RESULTS

### Descriptive analyses

Our study collected 4364 effective responses, representing everyone who responded with an average of 30.95 (accounting for 85.972% of the overall anticipated responses), in the mediating model and 3880 valid responses with an average of 31.04 (accounting for 86.222% of the overall anticipated responses) in the moderating model. Means, standard deviations, and intraclass correlation coefficients (ICCs) among the primary variables in the study are shown in Table 1. ICC(1) is the ratio of between‐group variance to total variance, and ICC(2) is the group‐mean reliability (LeBreton & Senter, 2008). The null models showed that the ICC(1) of state rumination and depression was .548–.607, indicating that 54.8%–60.7% of the variance was due to interindividual factors and 39.3%–45.2% was due to intraindividual factors; ICC(2) was greater than .70, implying that a multilayer linear model was appropriate (see Table 1).

**TABLE 1:** Descriptive statistics: Means and *SD*s.

Variables	*N*	*M*	*SD*	ICC(1)	ICC(2)
141 participants (mediation model)					
Daily perceived stress	4364	38.64	25.11		
State rumination	4364	2.88	1.73	.546	.974
Depression	4364	15.34	22.10	.598	.978
125 participants (moderation model)					
Daily perceived stress	3880	37.89	24.32		
Depression	3880	14.65	21.48	.607	.979
Trait rumination	125	51.16	13.20		

Abbreviation: ICC, intraclass correlation coefficient.

### Effect of daily perceived stress on depression

Following the correlation results, we performed multilevel regression analyses to further investigate the predictive role of daily perceived stress on depression in daily life.

**FIGURE 2:**
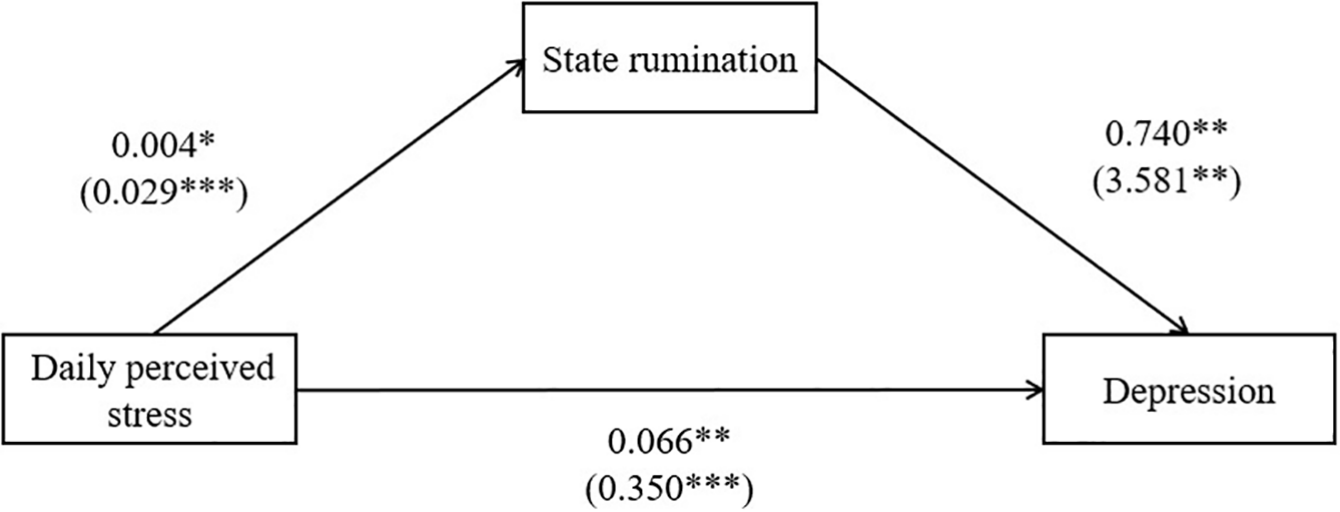
Mediation findings between daily perceived stress and depression. All parameters represent regression coefficients. The regression coefficients at the intraindividual level are outside parentheses, and those at the interindividual level are in parentheses. *p < .05; **p < .01; ***p < .001.

In the first step (Figure 2 and Table 3), when individuals perceived more daily stress at time *t*, they experienced more depression at time *t* + 2 (*B*_10(depression)_ = 0.069, *SE* = 0.025, *p = *.007). At the interindividual level of analysis, the results indicated that individuals with higher levels of perceived stress also had higher levels of depression (*B*_01(depression)_ = 0.456, *SE* = 0.095, *p < *.001).

### The mediation effect of state rumination

As daily perceived stress positively predicted depression, we tested the mediation effect of rumination between perceived stress and depression at both the inter and intraindividual levels.

The second step (Figure 2 and Table 2) found that the greater the daily perceived stress at time *t*, the higher the state rumination at time *t* + 1 (*B*_10(state rumination)_ = 0.004, *SE* = 0.002, *p* = .022) at the intraindividual level. The results also indicated that the higher the perceived stress, the greater the rumination (*B*_01(state rumination)_ = 0.029, *SE* = 0.005, *p* < .001) at the interindividual level.

**TABLE 2:** Model 2 for the mediating effect of state rumination and overall rumination.

Dependent variable	Level	Model 2 (daily perceived stress)		
		*B*	*SE*	*p*
State rumination	1	0.004	0.002	.022
	2	0.029	0.005	<.001

*Note*: *B* is the regression coefficient, and *p* is statistical significance. Level 1: Parameters of dynamic independent variables. Level 2: Parameter of the mean of dynamic independent variables on each subject.

The third step supported that state rumination at *t* + 1 positively predicted depression at *t* + 2 (Figure 2 and Table 3; *B*_20(depression)_ = 0.740, *SE* = 0.247, *p* = .003). After controlling state rumination, daily perceived stress at time *t* still significantly predicted depression at time *t* + 2 (*B*_10(depression)_′ = 0.066, *SE* = 0.025, *p* = .009). These results verified that state rumination could partially mediate the dynamic impact of daily perceived stress on depression. At the interindividual level, rumination positively predicted depression (*B*_02(depression)_ = 3.581, *SE* = 1.320, *p* = .008). After adding the average of state rumination, the perceived stress still positively predicted depression (*B*_01(depression)_′ = 0.350, *SE* = 0.095, *p* < .001).

**TABLE 3:** Model 1 and Model 3 of the mediating effect of state rumination and overall rumination.

	Level	Model 1 (daily perceived stress)			Model 3 (daily perceived stress and state rumination)					
		Daily perceived stress			Daily perceived stress			State rumination		
		*B*	*SE*	*p*	*B*	*SE*	*p*	*B*	*SE*	*p*
Depression	1	0.069	0.025	.007	0.066	0.025	.009	0.740	0.247	.003
	2	0.456	0.095	<.001	0.350	0.095	<.001	3.581	1.320	.008

*Note:* Level 1: Parameters of the dynamic independent variable and mediator; Level 2: Parameters of the mean of the dynamic independent variable and mediator on each subject.

In summary, the perceived stress of individuals could predict depression directly and impact it through two indirect paths. The first indirect path of perceived stress on depression was mediated by state rumination and accounted for 4.48% of the total variance of depression, which corroborated our hypothesis that state rumination may play a mediating role between daily perceived stress and depression at the intraindividual level. The second indirect effect was produced by overall rumination, which accounted for 29.67% of the total variance of overall depression, and this result proved that, at the interindividual level, state rumination also partially mediated the relationship between daily perceived stress and depression.

### The moderating role of trait rumination

In the moderating analysis, the first step investigated the dynamic influence of daily perceived stress at time *t* on depression at time *t* + 1, and the results were consistent (*B*_10(STRESS)_ = 0.077, *SE* = 0.024, *p* = .002), similar to the mediation model.

**FIGURE 3:**
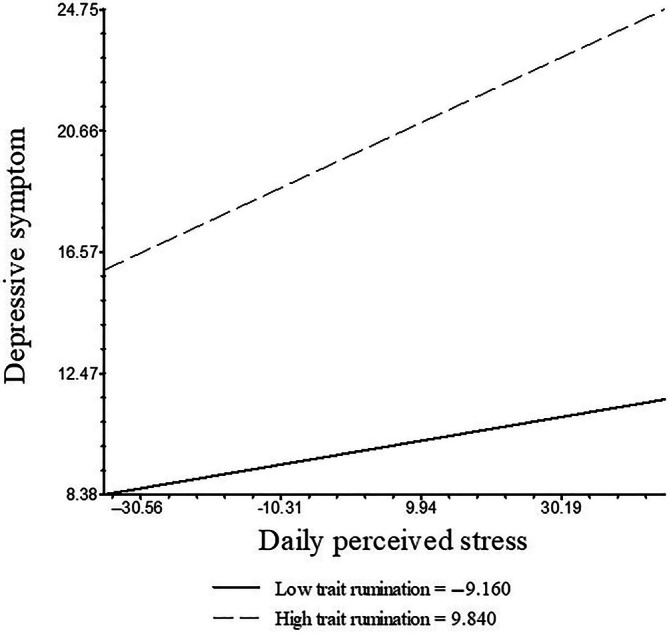
The simple slope analysis of the moderation role of trait rumination.

Subsequently, trait rumination was added as a moderator into the equation, and the results showed that college students scoring higher on trait rumination had more depression (*B*_01(depression)_ = 0.529, *SE* = 0.143, *p* < .001). The core parameters showed that college students scoring higher on trait rumination had significantly increased stress‐induced depression (*B*_11(depression)_ = 0.004, *SE* = 0.002, *p* = .048). Therefore, we concluded that trait rumination can moderate the relation of daily perceived stress at time *t* and depression at time *t* + 1. The simple slope analysis is shown in Figure 3. When trait rumination was at the 25th percentile (low trait rumination), the simple slope was 0.040 (*SE* = 0.031, *t* = 1.301, *p* = .196); when trait rumination was at the 75th percentile (high trait rumination), the simple slope was 0.109 (*SE* = 0.029, *t* = 3.796, *p* < .001).

## DISCUSSION

In this study, we employed an EMA design to examine the dynamic effects of stress perception on depression across daily life situations at both the interindividual and intraindividual levels and to test the mediating and moderating effects of rumination. The results demonstrated that daily stress perception directly affects depression and also indirectly affects it through the mediating role of state rumination, across various daily situations and over time. Meanwhile, trait rumination was found to moderate the relationship between daily perceived stress and depressive emotions.

Multilevel analysis results suggested that at both the inter and intraindividual levels, increased daily perceived stress would lead to more severe depression at the subsequent moment. This finding supports Hypothesis 1 of our study and is consistent with the results from previous cross‐sectional and long‐term longitudinal studies (Cristóbal‐Narváez et al., 2020; Mayer et al., 2018). It is also in line with the diathesis‐stress theoretical framework, which posits that stress factors leading to depression (Monroe & Simons, 1991) depend on stress perception (Novais et al., 2017). Changes in the living environment, academic pressure, and interpersonal problems are prevalent stressors among college students. If they cannot cope with or adapt well to the stressors, they may develop psychological issues, particularly depression (Ota et al., 2020).

The present study also demonstrated the mediating effect of state rumination on the dynamic impact of perceived stress on depression in natural contexts, thus supporting Hypothesis 2. Previous work has found that state rumination mediates the relationship between negative affect and binge eating (Smith et al., 2021). The current study extends this understanding by exploring the mediating role of trait rumination in the context of depression. These results indicated that, on the one hand, stress perception positively predicted the temporal changes in college students' state rumination, meaning that higher perceived stress would lead to increased state rumination (Connolly & Alloy, 2017). On the other hand, state rumination at one moment can trigger more depression at the next moment, indicating that a negative cognitive reaction style can intensify or prolong the duration of individuals' depression (Rosenbaum et al., 2021). This mechanism is an important cognitive pathway in the formation of depression (Nolen‐Hoeksema et al., 2008). These findings are in line with the cognitive activation theory of stress, suggesting that it is not brief stress itself that directly gives rise to the negative psychological and physiological activation but is an indirect way through the individual's negative outcome expectations caused by the cognitive assessment of the stress (Ursin & Eriksen, 2010). In other words, college students may experience depressive symptoms not solely because of daily stress but also due to negative outcome expectations, such as state rumination, when they perceive a lack of control or influence over the stress outcome.

In addition, trait rumination can also significantly moderate the dynamic effect of perceived stress on depression, thus supporting Hypothesis 3. Specifically, for individuals with high levels of trait rumination, the predictive effect of daily stress perception on depression is significantly enhanced. Although many studies have examined the role of rumination in intensive longitudinal research (Hjartarson et al., 2021), we innovatively treated trait rumination as an interindividual variable, exploring individual heterogeneity in the intensive dynamic pathways to depression. The catalytic role of trait rumination supports prior research suggesting that it is a risk mechanism for mental health (Hsu et al., 2015). The attentional scope model of trait rumination explains that restricted thinking, perception, and behavior can influence executive functions in individuals with high trait rumination (Whitmer & Gotlib, 2013). This functional impairment may lead individuals to confine their attention to negative information, resulting in a range of negative experiences (Liu et al., 2021). Conversely, college students with low levels of trait rumination might employ positive coping styles when perceiving stress. These positive coping styles help eliminate negative thoughts, thereby maintaining positive emotional experiences later (Zheng et al., 2020). Our findings also align with the diathesis‐stress model, which posits that the combination of life event‐induced stress with negative reasoning style, cognitive bias, and rumination is a risk factor for depression (Monroe & Simons, 1991). In conclusion, these moderating results support the risk‐shaping hypothesis of trait rumination and extend its applicability to daily life.

This study offered insights into mental health practice. Results suggested that daily life perceived stress can significantly predict immediate future depression, particularly in individuals with high levels of trait rumination. First, the study has shed light on the need for school mental health facilities to monitor and identify daily stress in college students rather than merely emphasize the stress resulting from major life events, as daily perceived stress can exacerbate depressive symptoms through negative thinking. As empirical studies and meta‐analyses have found the effectiveness of school‐based stress management interventions, incorporating evidence from natural contexts, such as EMA, can enhance these interventions to help students better cope with stress in their daily life environment (Johnstone et al., 2016; van Loon et al., 2020). Recently, due to the rapid development of e‐health, intervention techniques integrated with smart wearable devices or apps will facilitate a promising future to provide feasible, accessible, and affordable training programs for college students struggling with daily stress (Zhou et al., 2023). Second, addressing daily rumination among college students is crucial for its role in the occurrence of daily life depressive experiences. Mindfulness practice, proven to effectively intervene in rumination and stress perception (Gu et al., 2018), and attentional control related to depressive symptoms via rumination (Hsu et al., 2015), can be implemented. Furthermore, a 6‐day ecological momentary intervention with twice‐daily exposure to natural images has been shown to reduce stress and rumination (Beute & de Kort, 2018). In the future, mental health practitioners should be encouraged to regard daily state rumination as a vital target because it mediates the relationship between daily perceived stress and depression. As a beneficial supplement, a cognitive‐behavioral therapy (CBT) self‐help program focused on rumination can have a therapeutic effect on rumination, worry, and anxiety, which could be guided with minimal instruction and can be complemented with clinical practice and psycho‐education (Umegaki et al., 2022). College students facing daily stress and state rumination can select the most suitable intervention programs based on accessibility and digital literacy. Third, our findings underscore the importance of considering trait rumination as a mental health risk, as students with higher levels of this trait are more vulnerable to depression when experiencing daily stress. Special attention is warranted when such students face high daily stress levels.

Although the present study produced some innovative results, it still has some limitations. First, the study participants were healthy college students; hence, caution must be exercised when extending the conclusions to other groups. Second, the data collection was based on self‐reports. Although this approach has been validated in previous research, future studies would benefit from incorporating other objective physiological indices (e.g., cortisol secretion) during data collection to offer more robust model testing. Finally, more connotations or multiple dimensions of depression should be fully considered when measuring depression to improve the validity of the study. Additionally, future studies should explore the presence of other mediating or moderating factors in the impact of perceived stress on depression.

## CONCLUSION

With the EMA approach, this study offered a more comprehensive understanding of the temporal dynamics between perceived stress and depression in daily life. Daily perceived stress was found to directly affect depressive symptoms in college students and indirectly through the critical mediating mechanism of state rumination. Additionally, a high level of trait rumination is a risk factor that exacerbates the effect of daily perceived stress on depression among college students.

## CONFLICT OF INTEREST STATEMENT

We declare that we do not have any commercial or associative interest that represents a conflict of interest in connection with the work submitted.

## ETHICS STATEMENT

This study involving human participants were reviewed and approved by the Ethics Committee of Southwest Jiaotong University. The participants provided their written informed consent to participate in this study.

## Supporting information


**Data S1.** Supporting information.
