# Supplementary material for: The temporal dynamics of perceived stress and depression in college students: An ecological momentary assessment
Source: Psych J. 2024 Oct 4;14(4):491–9. doi: 10.1002/pchj.803 (PMC12318596; doi:10.1002/pchj.803)
Supplement: Supplementary file 1 — Data S1. Supporting information. [file PCHJ-14-491-s001.docx]

Supplementary Materials

***Data analysis***

*1. The mediation effect*

Hierarchical linear models have been increasingly used in longitudinal studies (Singer & Willett, 2003). This statistical approach enables the exploration of the effects of variables at different levels on dependent variables. We followed the multilevel mediation approach of “centered within the context with the reintroduction of the subtracted means” to test the mediation hypotheses. Due to all of the variables in the mediation model being measured at Level 1 (within-person), we constructed a 1-1-1 multilevel mediation model using HLM 7.0. In this model, the dependent variable was daily depression, the first level was intra-individual data (daily perceived stress, state rumination), and the second level was inter-individual data that were the mean of dynamic variables on each subject (overall perceived stress, overall rumination). Aligned to the work of Du et al. (2019), our study used three steps to test the mediation process between daily perceived stress and depression as follows:

First, the equation between the independent variable (daily perceived stress) and the dependent variable (depression) was established in Level 1. Meanwhile, the average stress measured by each participant over 12 days was calculated and added to Level 2. Model 1 was obtained:

MODEL 1

Level 1:

Depression_(t+1) i_=π_0i_+π_1i_*(STRESS_ti_) + e_ti_

Level 2:

π_0i_=b_00_+b_01_*(M_i__STRESS) + u_0i_

π_1i_=b_10_

In the above equation, *t* represented the moment, and *i* represented the individuals. Depression*_(t+1)i_* represented the depression of the *i*th subject at time *t + 1*, and *STRESS_ti_* represented the perceived stress of the *i*th subject at time *t*. *MI_STRESS* was the average of all the stress measured by the *i*th subject, that represented the total stress of the subject. The intercept π*_0i_* represented the mean value of depression measured by the *i*th subject. *e_ti_* stands for residual. *b_10_* represented the predictive degree of perceived stress at time t to depression at time *t* + 1, and *b_01_* represented the predictive effect of perceived stress on depression in general.

The second step was to establish the relationship between the independent variable (daily perceived stress) and the dependent variable (state rumination) at Level 1. We then averaged the daily perceived stress for each individual and put it into Level 2 in the equation, as follows:

MODEL 2

Level 1:

RUM_(t+1) i_=π_0i_+π_1i_*(STRESS_ti_) +e_ti_

Level 2:

π_0i_=b_00_+b_01_*(M_i__STRESS) +u_0i_

π_1i_=b_10_

Similar to the first equation, *RUM_(t+1)i_* represented the degree of self-rumination of the *i*th subject at the time *t+1*. In this model, *b_10_* represented the prediction degree of perceived stress at one moment to state rumination at the next moment on the intra-individual level, and *b_01_* represented the prediction effect of stress on a rumination at the inter-individual level.

In the third step, the relationship between the independent variable (daily perceived stress), the mediating variable (state rumination), and the dependent variable (depression) is established at Level 1. The means of the independent variable (perceived stress) and mediating variable (rumination) for each individual were calculated and placed in Level 2 as seen in MODEL 3:

MODEL 3

Level 1:

Depression_(t+1) i_=π_0i_+π_1i_*(STRESS_ti_)+π_2i_*(RUM_(t+1) i_) +e_ti_

Level 2:

π_0i_=b_00_+b_01_*(Mi_STRESS) +b_02_*(Mi_RUM) +u_0i_

π_1i_=b_10_

π_2i_=b_20_

The symbols in this equation were mostly the same as above. *Mi_RUM*, which had not appeared before, represented the rumination. In this equation, *b_10_* and *b_20_* respectively represented the predictive effect of daily perceived stress at a particular moment and state rumination at the next moment on depression, while *b_01_* and *b_02_* respectively represented how much the perceived stress and rumination could predict depression at the inter-individual level.

*2. The moderating effect*

Multilevel linear models have been used to explore the moderating effect of trait variables (Xu et al., 2017). To examine the moderating role of trait rumination, we developed another multilevel linear model with daily perceived stress and depression measured by dynamic assessments and trait rumination measured via RRS. The equation is the same as that used in the first step of the mediating effect test for level 1:

Depression_(t+1)i_=π_0i_+π_1i_*(STRESS_ti_)+e_ti_

A level 2 equation was established to investigate whether trait rumination moderates the relationship between daily perceived stress and depression.

π_0i_=b_00_+b_01_*(RUM_i_)+u_0i_

π_1i_=b_10_+b_11_*(RUM_i_)+u_1i_

In the above two equations, *b_00_* represented the average depression of *i*th subject. *b_10_* represented the predictive power of the stress at time *t* to the depression at *t+1*. Additionally, *b_01_* represented the predictive power of trait rumination for depression, and *b_11_* represented the influence of trait rumination on the relationship between daily perceived stress at time *t* and depression at time *t+1*. That is to say, whether rumination moderates the association between daily perceived stress and depression depends on whether b11 is significant.
